# Potential Roles of Exercise-Induced Plasma Metabolites Linking Exercise to Health Benefits

**DOI:** 10.3389/fphys.2020.602748

**Published:** 2020-12-03

**Authors:** Yong Ryoul Yang, Ki-Sun Kwon

**Affiliations:** ^1^Aging Research Center, Korea Research Institute of Bioscience and Biotechnology, Daejeon, South Korea; ^2^Department of Functional Genomics, KRIBB School of Bioscience, Korea University of Science and Technology (UST), Daejeon, South Korea

**Keywords:** exercise, metabolites, alpha-ketoglutarate (α-KG), beta-aminoisobutyric acid (BAIBA), kynurenic acid (KYNA), β-hydroxybutyrate (BHB), lactate, 12,13-diHOME

## Abstract

Regular exercise has a myriad of health benefits. An increase in circulating exercise factors following exercise is a critical physiological response. Numerous studies have shown that exercise factors released from tissues during physical activity may contribute to health benefits *via* autocrine, paracrine, and endocrine mechanisms. Myokines, classified as proteins secreted from skeletal muscle, are representative exercise factors. The roles of myokines have been demonstrated in a variety of exercise-related functions linked to health benefits. In addition to myokines, metabolites are also exercise factors. Exercise changes the levels of various metabolites *via* metabolic reactions. Several studies have identified exercise-induced metabolites that positively influence organ functions. Here, we provide an overview of selected metabolites secreted into the circulation upon exercise.

## Introduction

**Figure 1:**
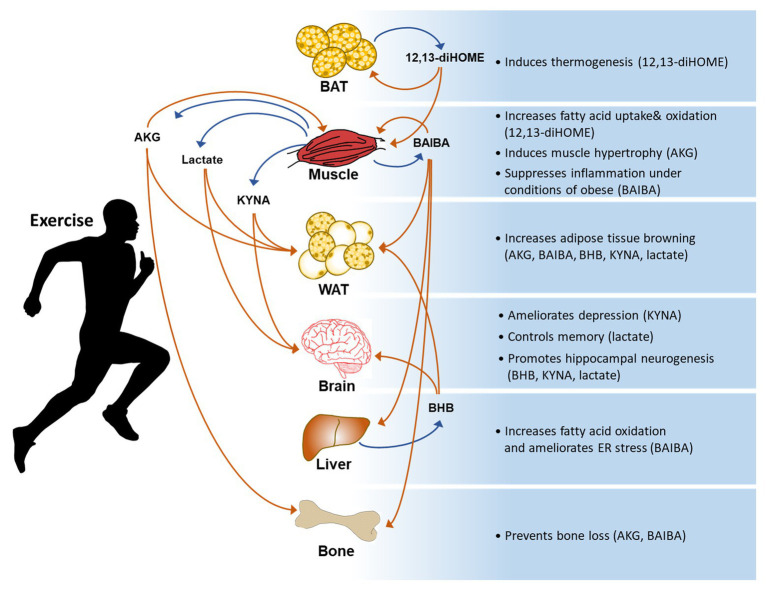
A general summary of exercise-induced metabolites and their effects on the body. AKG: α-ketoglutaric acid, BAIBA: β-aminoisobutyric acid, BHB: β-hydroxybutyrate, KYNA: kynurenic acid, and 12,13-diHOME: 12,13-dihydroxy-9Z-octadecenoic acid.

Exercise benefits every part of the body and prevents chronic diseases. The effects of exercise are mediated by a complex process involving interorgan crosstalk and activation of integrated body systems at the molecular, cellular, and systemic levels. However, the cellular and molecular mechanisms underlying the effects of exercise are unclear. Omics technologies have made it possible to obtain a huge number of molecular measurements within a tissue, a cell, or plasma during exercise and to comprehensively understand the effects of physical exercise (Pourteymour et al., 2017; Whitham et al., 2018; Pillon et al., 2020). Numerous studies have identified exercise factors that are part of a complex network of interorgan communication. Diverse tissues including skeletal muscle, adipose tissue, bone, and the liver release exercise factors into blood (Moon et al., 2016; Ingerslev et al., 2017; Zhang et al., 2017; Takahashi et al., 2019). These factors contribute to the beneficial effects of exercise, including reduction of adipose mass and inflammation, maintenance of muscle mass, improvement of cardiovascular fitness, and promotion of brain plasticity, as discussed in many previous studies (Hawley et al., 2014). Various types of exercise change whole-body metabolism in both clinical and animal models (Huffman et al., 2014; Starnes et al., 2017; Sato et al., 2019; Schranner et al., 2020). Metabolism plays a crucial role in human health and disease, and is modulated by intrinsic and extrinsic factors. Several exercise-induced metabolites mediate metabolic functions including thermogenesis, glucose homeostasis, and lipolysis (Roberts et al., 2014; Stanford et al., 2018; Yuan et al., 2020). The list of novel metabolites released by exercise continues to grow, aided by advanced omics technologies. However, the roles of many metabolites remain to be tested in murine and human *in vivo* models. Elucidation of the mechanism underlying interorgan crosstalk and biological networking involving exercise factors will help to identify potential therapeutic targets. In this review, we selected six metabolites that have been extensively characterized and have therapeutic potential in metabolic disorders, neurodegenerative diseases, osteosarcoma, or sarcopenia. We summarize our current knowledge of these metabolites, focusing on their biological functions (Figure 1, Table 1).

## α-Ketoglutaric Acid

α-Ketoglutaric acid (AKG) is a crucial intermediate in the TCA cycle required for a rate limiting step (Krebs and Johnson, 1980). AKG is involved in various types of cellular energy metabolism and a variety of metabolic pathways. AKG can be decarboxylated to succinyl-CoA and CO_2_ by AKG dehydrogenase in the TCA cycle. In addition, AKG is generated from isocitrate by oxidative decarboxylation catalyzed by isocitrate dehydrogenase or anaplerotically from glutamate by oxidative deamination using glutamate dehydrogenase. AKG is a key metabolite in the TCA cycle and is therefore mostly present in mitochondria and the cytoplasm of cells. In addition, AKG is also found in blood (Rocchiccioli et al., 1984; Wagner et al., 2010). Yuan et al. (2020) recently analyzed serum metabolites after acute resistance exercise and found that TCA cycle intermediates are upregulated. Notably, the serum level of AKG is significantly elevated in response to exercise. In humans, physical exercise increases serum levels of AKG (Lewis et al., 2010; Brugnara et al., 2012). AKG elicits exercise-induced beneficial effects, including muscle hypertrophy and fat loss, through 2-oxoglutarate receptor 1 (OXGR1)-dependent adrenal activation (Yuan et al., 2020). Several studies have shown that AKG activates the mammalian target of rapamycin (mTOR) signaling pathway, resulting in protein synthesis and skeletal muscle hypertrophy (Yao et al., 2012; Cai et al., 2016). AKG inhibits protein degradation and skeletal muscle atrophy through a prolyl hydroxylase 3 (PHD3)/β2 adrenergic receptor (ADRB2)-mediated mechanism (Cai et al., 2018). In addition to skeletal muscle, AKG has positive effects on several tissues. AKG injection protects against bone loss in ovariectomized rats (Radzki et al., 2012). AKG supplementation promotes beige adipogenesis through AKG-mediated demethylation in the *Prdm16* promoter (Tian et al., 2020). These beneficial effects induced by AKG resemble several changes induced by resistance exercise (Lesinski et al., 2016). These studies suggest that exercise-induced AKG partially contributes to the metabolic effects of exercise.

## β-Aminoisobutyric Acid

β-Aminoisobutyric acid (BAIBA) is a non-proteinogenic amino acid. It is a natural catabolite of thymine and valine metabolism in mammals. There are two enantiomers of BAIBA in biological systems: D-BAIBA (R-BAIBA) and L-BAIBA (S-BAIBA). L-BAIBA is produced *via* catabolic reactions of L-valine, while D-BAIBA is generated in the cytosol as an intermediate product of thymine degradation (Fink et al., 1956; Kupiecki and Coon, 1957). However, the systemic regulation of their levels is not clearly understood. BAIBA is an exercise-responsive metabolite. Roberts et al. (2014) found that chronic exercise and muscle-specific peroxisome proliferator-activated receptor (PPAR) γ coactivator 1α (PGC-1α) overexpression increase the plasma level of BAIBA in mice. Stautemas et al. (2019) found that acute aerobic exercise increases the plasma levels of D-BAIBA and L-BAIBA by 13 and 20%, respectively, and that alanine-glyoxylate aminotransferase 2 (AGXT2) polymorphism only affects the plasma level of D-BAIBA. BAIBA increases expression of brown adipocyte-specific genes in primary adipogenic precursor cells and induces browning in white adipose tissue (WAT) through a PPARα-dependent pathway. BAIBA also increases hepatic fatty acid β-oxidation. These effects of BAIBA on adipose and liver tissues reduce weight gain and improve glucose tolerance in mice (Roberts et al., 2014). Consistently, BAIBA protects against obesity and related metabolic disorders in mice with partial leptin deficiency (Begriche et al., 2008). In type 2 diabetes model mice, BAIBA ameliorates hepatic endoplasmic reticulum stress, apoptosis, and glucose/lipid metabolic disturbance (Shi et al., 2016). In addition, BAIBA improves palmitate‐ and high-fat feeding-induced insulin resistance and inflammation, *via* its action on AMP-activated protein kinase (AMPK) and PPARδ in skeletal muscle (Jung et al., 2015). In addition to its metabolic effects, L-BAIBA prevents reactive oxygen species (ROS)-induced apoptosis of osteocytes and loss of bone and muscle. The effects of L-BAIBA are mediated *via* Mas-related G protein-coupled receptor type D (MRGPRD), and reduced MRGPRD expression with age results in loss of the protective effect of L-BAIBA (Kitase et al., 2018). BAIBA also ameliorates fibrotic responses and renal functional impairment in obstructed kidneys by downregulating the angiotensin (Ang) II/interleukin (IL)-17/ROS signaling pathway (Wang et al., 2017). These reports suggest that BAIBA affects diverse tissues as a mediator of the beneficial effects of exercise.

## Kynurenic Acid

Tryptophan is not only an α-amino acid used to biosynthesize proteins, but is also a precursor of many biologically active compounds such as serotonin, melatonin, and indole. The main route for tryptophan catabolism is through the kynurenine (KYN) pathway. In this pathway, a product of one branch is kynurenic acid (KYNA) and the final product is nicotinamide adenine nucleotide (NAD+). KYNA is generated in diverse types of peripheral tissues and cells, and is also present in various products consumed by humans. The KYN pathway is responsible for over 90% of peripheral tryptophan metabolism (Leklem, 1971). KYN and its metabolites are involved in many fundamental biological and pathophysiological processes. Agudelo et al. (2014) found that overexpression of PGC-1α1 in muscle (mck-PGC-1α1) elevates expression of KYN amino transferases (KATs) 1, 2, and 3, and that exercise training increases KAT expression in skeletal muscle. Importantly, the plasma KYNA level is increased in exercise-trained mice. Interestingly, mck-PGC-1α1 transgenic mice are resistant to depression. Agudelo et al. suggested that elevated peripheral conversion of KYN to KYNA in skeletal muscle blocks accumulation of neurotoxic KYN metabolites that contribute to the pathogenesis of depression in the brain. Consistently, Schlittler et al. (2016) showed that endurance exercise, but not resistance exercise, increases the plasma KYNA level. In addition to depression, accumulation of KYN and its metabolites in the central nervous system is associated with several psychiatric disorders (Miller et al., 2006; Sellgren et al., 2016). In the brain, KYNA is an antagonist of N-methyl-D-aspartate (NMDA) receptors, α-amino-3-hydroxy-5-methyl-4-isoxazolepropionic acid receptors, kainate receptors, and α7 nicotinic acetylcholine receptor (α7nAChR; Perkins and Stone, 1982; Hilmas et al., 2001). By contrast, KYNA acts as an agonist of the G-protein-coupled receptor GPR35 (Wang et al., 2006). Furthermore, KYNA has antioxidant properties (Lugo-Huitron et al., 2011; Perez-Gonzalez et al., 2015). Although the underlying molecular mechanism remains unclear, accumulating evidence suggests that KYNA has neuroprotective properties and there is great therapeutic potential in targeting the muscle KAT-KYN pathway in psychiatric disorders. In addition to the brain, Agudelo et al. (2018) found that KYNA functions as a ligand of GPR35 in adipose tissue. A single daily dose of KYNA, which elevates its plasma level to that observed upon exercise, increases systemic energy expenditure through GPR35, which enhances expression of lipid metabolism, thermogenic, and anti-inflammatory genes in adipose tissue. These reports suggest that the beneficial effects of exercise on the brain and adipose tissue might be attributable to an increased level of circulating KYNA.

## β-Hydroxybutyrate

Ketone bodies are small lipid-derived molecules produced by the liver during fasting and upon prolonged exercise. They are distributed *via* the circulation to peripheral tissues including skeletal muscle and the brain, where they can be converted to acetyl-CoA (Newman and Verdin, 2014). β-Hydroxybutyrate (BHB) is the most prevalent ketone body in mammals and plays pivotal roles in whole-body energy metabolism. Much evidence suggests that BHB is a biologically active metabolite with a broad range of signaling and regulatory effects. BHB is an inhibitor of histone deacetylases (HDACs) and its plasma level is increased after a single bout of acute exercise in mice and human (Kim et al., 2013). Sleiman et al. (2016) revealed that exercise induces brain-derived neurotrophic factor (BDNF) expression in the hippocampus *via* the action of BHB. They found that BHB released in response to exercise induces BDNF expression by inhibiting HDACs and increases in neurotransmitter release in hippocampus. BDNF has positive effects on memory, cognition, and synaptic transmission. Thus, it is highly conceivable that BHB can enhance plasticity and improve cognition through BDNF expression. Accumulating evidence suggest that HDAC inhibitors can improve cognitive impairment resulting from neurodegenerative disorders (Graff and Tsai, 2013). Similarly, a recent report revealed that BHB improves cognitive function in 5XFAD mouse, a widely used AD mouse model, by attenuating Aβ accumulation and microglia overactivation (Wu et al., 2020). BHB elicits neuroprotective effects against hypoxic and hypoglycemic insults and N-methyl-D-aspartate-induced excitotoxicity (Masuda et al., 2005; Samoilova et al., 2010). BHB attenuates neuroinflammation pathology by inhibiting NLRP3 inflammasome activation in Alzheimer’s disease or the spinal cord injury model (Kong et al., 2020; Shippy et al., 2020). Also, BHB prevents post-sepsis cognitive impairment (Wang et al., 2020). These multiple studies suggest that BHB may be beneficial in preventing neurodegenerative diseases.

It is noteworthy that butyrate, a broad HDAC inhibitor, improves insulin sensitivity and increases the metabolic rate and oxidative metabolism in a mouse diabetes model (Galmozzi et al., 2013). Thus, the metabolic effects of exercise may be partially attributable to BHB-induced adipocyte browning through a change in the intracellular redox state (Carriere et al., 2014). Ketogenic diets increase the levels of circulating BHB. Ketogenic diets, which contain much fat and little carbohydrate, elevate uncoupling protein 1 (UCP1) expression in brown adipose tissue (BAT) of mice and reduce body weight (Kennedy et al., 2007; Moreno et al., 2014). Additionally, ketogenic diets increase the exercise capacity and show a preventive effect on organ injury caused by acute exercise in mice despite the decrease of absolute muscle volume (Ma et al., 2018). Accumulating evidence indicate that the observed benefit of ketogenic diets might be attributed elevation of circulating BHB. Of note, circulating BHB is also elevated during caloric restriction (CR) or fasting. CR is widely accepted as positive control of anti-aging intervention (Fontana et al., 2010; Swindell, 2012). BHB has been proposed as a mediator of the beneficial anti-aging effects associated with CR. BHB extends the lifespan of *C. elegans* through inhibiting HDACs and the DAF16/FOXO and SKN-1/Nrf pathways (Edwards et al., 2014). Han et al. (2018) found that BHB prevents p53 independent and octamer-binding transcriptional factor (Oct) four dependent senescence in mouse vascular cells. BHB upregulates Oct4 expression *via* interacting with heterogeneous nuclear ribonucleoprotein A1 (hnRNP A1), inducing cell quiescence. Intraperitoneal injection of BHB alleviates vascular aging in mice (Han et al., 2018). Because of the BHB roles, it can be considered as a potential mediator of the anti-aging effects of CR and exercise. Recently, numerous studies have shown that BHB is involved in a variety of cellular functions. BHB promotes generation of claudin-5 and attenuates diabetes-associated cardiac microvascular hyperpermeablility by inhibiting HDAC3 (Li et al., 2020). In aged mice, BHB ameliorated hepatic ER stress and lipid accumulation through the GPR109A/AMPK pathway (Lee et al., 2020). BHB supplementation has also been shown to improve exercise capacity by altering mitochondrial morphology and functions (Monsalves-Alvarez et al., 2020). Taken together, these studies suggest that BHB released upon physical exercise contributes to a wide-range of positive health effects *via* a number of potential cellular mechanisms.

## Lactate

Lactate is the ultimate final product of anaerobic glycolysis. Anaerobic exercise induces conversion of pyruvate into lactate by lactate dehydrogenase. Lactate is not metabolized further and is released into blood. Almost 40% of lactate in the circulation is generated by skeletal muscle (Juel et al., 1990; Adeva-Andany et al., 2014; Lonbro et al., 2019). Maximal exercise can cause a ∼20-fold increase in circulating lactate (Goodwin et al., 2007). Many reports have shown that lactate is a signaling molecule released from muscle to communicate with other tissues, such as the brain, the liver, adipose tissue, and the heart (Brooks, 2009). De Matteis et al. (2013) reported that exercise strongly induces expression of the lactate importer MCT1 and increases the metabolic activity of brown adipocytes and suggested that lactate metabolism controls these cells. Carriere et al. (2014) tested the effect of lactate on browning of subcutaneous WAT (scWAT). They found that an increase in browning correlates with an increase in circulating lactate and MCT1 expression in scWAT. Lactate increases thermogenic gene expression in adipocytes *via* PPARδ, and administration of lactate induces browning in scWAT of mice. Uptake and metabolism of lactate have been demonstrated in the brain. Lactate uptake by neurons correlates with an increase in the plasma lactate level. Interestingly, lactate use by the brain is related to neuronal activity (Serres et al., 2004; Kemppainen et al., 2005; Dalsgaard, 2006). Lev-Vachnish et al. (2019) identified L-lactate as a factor that promotes adult hippocampal neurogenesis. L-lactate enters neurons through MCT2 and induces formation of new neurons in the dentate gyrus. In addition to neurons, astrocytes regulate memory formation by controlling neuronal lactate transport (Suzuki et al., 2011). Several studies have suggested that increased vascular density is critical for maintaining cognitive function in the brain (Ding et al., 2006; Wightman et al., 2015). Morland et al. (2017) demonstrate that exercise increases brain vascular endothelial growth factor A (VEGFA) protein and angiogenesis *via* the lactate receptor HCAR1. Moreover, lactate controls blood flow in the brain by increasing vasodilation to obtain more oxygen and glucose when the oxygen concentration is low (Gordon et al., 2008). A number of recent studies implicate the role of lactate in the control of energy intake in rodents and humans. Of note, lactate inhibits both production and activation of ghrelin in gastric mucosal cells (Engelstoft et al., 2013). Lactate also influences appetite through modulating hypothalamic neuropeptide expression and release (Cha and Lane, 2009; Ou et al., 2019). Taken together, these data indicate that lactate is an important metabolic product and that circulating lactate may mediate the beneficial effects of exercise on metabolism and cognition.

## 12,13-Dihydroxy-9Z-Octadecenoic Acid

Oxylipins are oxidized metabolites of long-chain polyunsaturated fatty acids (PUFAs). PUFAs can be obtained directly from the diet or from metabolism of linoleic acid and α-linolenic acid. Oxylipins are detected in all tissues, urine, and blood (Gabbs et al., 2015). Imbalances in oxylipins correlate with pathological conditions including metabolic disorders, depression, pain, and cardiovascular disease (Caligiuri et al., 2017; Deol et al., 2017; Hennebelle et al., 2017). Linoleic acid can be metabolized *via* the cytochrome P450 (CYP) pathway to generate 12,13-diHOME. Lipidomics analysis demonstrated that the plasma 12,13-diHOME level increases in response to an acute bout of exercise in humans and mice (Stanford et al., 2018). Exercise-induced 12,13-diHOME is released from BAT. Acute treatment of mice with 12,13-diHOME increases skeletal muscle fatty acid uptake and oxidation (Stanford et al., 2018). Similar to exercise, cold exposure stimulates activation of BAT. Another lipidomic analysis revealed that the plasma 12,13-diHOME level is elevated following cold exposure in humans and mice. Injection of 12,13-diHOME facilitates BAT thermogenesis by selectively promoting fatty acid uptake, leading to enhanced cold tolerance. Chronic treatment of diet-induced obese mice with 12,13-diHOME protects against cold challenge and high-fat diet-induced obesity. These results indicate that lipid metabolites participate in regulation of metabolic changes in response to exercise.

## Conclusion

Exercise induces release of many regulatory factors into the circulation and these factors influence body changes. Studies have found many exercise factors that link exercise to beneficial effects. Myokines are representative exercise factors secreted by skeletal muscle and affect diverse peripheral tissues as mediators of interorgan crosstalk. Exercise induces great metabolic changes and release of biologically active metabolites into blood. Studies using diverse approaches have identified novel metabolites that link exercise to beneficial effects. Many exercise-induced factors including metabolites and myokines have been reviewed elsewhere (Rai and Demontis, 2016; Murphy et al., 2020).

Investigations have mainly focused on upregulated metabolites that influence tissue functions. Similar to exercise-induced metabolites, plasma metabolites whose levels are reduced during exercise may play important roles in regulating the beneficial effects of exercise. Indeed, the levels of many plasma metabolites increase with age and some of these metabolites might be associated with aging and age-related diseases (Darst et al., 2019; Yeri et al., 2019). It will be important to investigate the functional role of age-related metabolites whose levels are decreased by exercise. Contrepois et al. (2020) recently performed longitudinal profiling of blood metabolites before and after acute exercise in human and found 728 metabolites affected by exercise. Schranner et al. (2020) identified 196 metabolites that are significantly changed by endurance or resistance exercise in human. These data might be a useful resource to investigate mechanisms of exercise-induced health benefits. Recently, it has been observed that physical exercise modulates gut microbiota in both humans and animals (Clarke et al., 2014; Barton et al., 2018). Of note, bacteria-derived metabolites play critical roles in the modulation of aging and longevity in the host organism (Smith et al., 2017; Shin et al., 2020). Thus, microbiota-derived metabolites could be important mediators of the benefits of exercise. As exercise-induced metabolites have health benefits and reduce the risk of many diseases in humans, elucidation of the roles of exercise-related metabolites and the underlying mechanisms will help to identify novel therapeutic targets for metabolic diseases, including type 2 diabetes and obesity.

**Table 1:** Summary of plasma metabolites induced by exercise.

Metabolites	Classes of metabolites	Subjects	Exercise protocol	Main tissue(s) of origin	References
α-Ketoglutaric acid	A product of glycolysis	Mouse	Acute resistance exercise (ladder-climbing)	Skeletal muscle	Yuan et al., 2020
		Human	26.2-mile marathon		Brugnara et al., 2012
			Short-term intensive exercise		Lewis et al., 2010
β-Aminoisobutyric acid	A product of pyrimidine metabolism	Human	Short-term intensive exercise	Skeletal muscle	Stautemas et al., 2019
		Mouse	3 week free wheel running exercise		Roberts et al., 2014
Kynurenic acid	A product of tryptophan metabolism	Human	Endurance exercise (a 150-km road cycling time trial)	Skeletal muscle	Schlittler et al., 2016
		Mouse	8 weeks of free wheel running		Agudelo et al., 2014
β-Hydroxybutyrate	A product of the normal metabolism of fatty acid	Mouse	4 weeks of free wheel running	Liver	Sleiman et al., 2016
		Human	Acute exercise (Treadmill running)		Kim et al., 2013
Lactate	A product of anaerobic glycolysis	Human	Short-term intensive exercise	Skeletal muscle	Juel et al., 1990
		Mouse	Acute exercise (Treadmill running)		Lonbro et al., 2019
12,13-Dihydroxy-9Z-octadecenoic	A product of linoleic acid metabolism	Human	Acute exercise (cycle ergometer)	Brown adipose tissue	Stanford et al., 2018

## Author Contributions

YY and K-SK conceptualized, designed, and wrote the review. K-SK approved the final version of the review. All authors contributed to the article and approved the submitted version.

### Conflict of Interest

The authors declare that the research was conducted in the absence of any commercial or financial relationships that could be construed as a potential conflict of interest.
